# Elevated Blood Urea Nitrogen-to-Serum Albumin Ratio as a Factor That Negatively Affects the Mortality of Patients with Hospital-Acquired Pneumonia

**DOI:** 10.1155/2019/1547405

**Published:** 2019-06-16

**Authors:** Ding-Yun Feng, Yu-Qi Zhou, Xiao-Ling Zou, Mi Zhou, Hai-Ling Yang, Xiao-Xia Chen, Tian-Tuo Zhang

**Affiliations:** ^1^Department of Respiratory and Critical Care Medicine, Third Affiliated Hospital of Sun Yat-Sen University, Institute of Respiratory Diseases of Sun Yat-Sen University, Guangzhou, China; ^2^Department of Surgery Intensive Care Unit, Third Affiliated Hospital of Sun Yat-Sen University, Guangzhou, China; ^3^Department of Medical Record, Third Affiliated Hospital of Sun Yat-Sen University, Guangzhou, China

## Abstract

This study aimed to evaluate the factors that affect 30-day mortality of patients with HAP. The data used in this study were collected from all HAP occurred in our hospital between January 2014 and December 2017. A total of 1158 cases were included. 150 (13.0%) of whom died within 30 days. This reported mortality identified by the univariate Cox regression analysis is found to have been affected by the following factors: age greater than 70 years, presence of diabetes mellitus and chronic obstructive pulmonary disease, gastric tube intubation, administration of proton-pump inhibitor, blood albumin level less than 30 g/l, elevated neutrophil-to-lymphocyte ratio, antibiotics therapy in the preceding 90 days, intensive care unit (ICU) admission, blood lymphocyte count less than 0.8 × 10^9^/L, elevated blood urea nitrogen/albumin (BUN/ALB) level, and presence of multidrug-resistant (MDR) pathogens. In the second multivariate analysis, administration of proton-pump inhibitor, administration of antibiotics in the preceding 90 days, ICU admission, blood lymphocyte count less than 0.8 × 10^9^/L, elevated BUN/ALB level, and presence of MDR pathogens were still associated with 30-day mortality. The area under the receiver operating characteristic curves in the BUN/ALB predicting 30-day mortality due to HAP was 0.685. A high BUN/ALB was significantly associated with a worse survival than a low BUN/ALB (*P* < 0.001). Therefore, an elevated BUN/ALB level is a risk factor for mortality on patients with HAP.

## 1. Introduction

Nosocomial or hospital-acquired pneumonia (HAP) is a common infection associated with a prolonged hospital length of stay and high patient mortality, generally with symptom onset at 48 h or more after admission to the hospital, or within 14 days of discharge from the hospital [1]. In the United States, it is the second most common nosocomial infection, after urinary tract infections, with the incidence rate ranging from 0.5% to 1% [2]. HAP is also common in China with an incidence rate of 1.74%–6.4% [3, 4], as well as in Vietnam where it is the most common nosocomial infection in adult intensive care units (ICUs) [5, 6]. HAP is a common hospital-acquired infection with a prolonged hospital length of stay and high patient mortality [1]. HAP is associated with a mortality rate ranging from 20% to 60%, especially in the ICU [7–9]. The attributable mortality is probably higher in some specific populations such as patients with chronic obstructive pulmonary disease (COPD) [10] and psychiatric diseases [11]. Hence, it is important to assess the factors related to HAP to predict patient prognosis. Therefore, this study aimed to evaluate the factors that affect 30-day mortality of patients with HAP, in order to improve its prognosis in the future.

## 2. Materials and Methods

Data related to all incidence of HAP that occurred in the third affiliated hospital of Sun Yat-sen University, Guangdong, China, between January 2014 and December 2017 were collected. Variables of interest included age, gender, comorbidity, central venous catheter use, prior treatment, laboratory data, and 30-day mortality. All patients included were aged at least 18 years. The criteria for a diagnosis of HAP [2] included a new pulmonary infiltrate (occurring ≥48 h after admission) associated with at least one of the following: new or increased cough with or without purulent tracheobronchial secretion or new pathogenic bacteria isolated from sputum or tracheal aspirate culture with ≥10^4^ colony-forming units/ml, fever (>37.8°C) or hypothermia (<35.6°C), leukocytosis, left shift, or leucopenia based on local normal values. Cases of ventilator-associated pneumonia and acquired immunodeficiency syndrome and those with missing key data were excluded.

Pathogenic bacteria isolated from the clinical specimens were further characterized by conventional biochemical tests to identify the specific strain by using standard microbiologic methods [12]. Pathogenic organism susceptibility testing was conducted using the microdilution method (MicroScan System; Baxter Healthcare, West Sacramento, CA, USA), and the results were interpreted using the National Committee for Clinical Laboratory Standards guidelines published in 2012 (Clinical & Laboratory Standards Institute, 2012). Here, multidrug-resistant (MDR) pathogens were defined as organisms resistant to at least one agent of three or more antimicrobial categories in susceptibility tests of isolates from patients with HAP [13].

### 2.1. Statistical Analysis

The 30-day survival was the main endpoint of this study. Survival was defined as the time interval between HAP diagnosis and death or the last follow-up. Multivariate analysis using a stepwise Cox proportional hazards model was used to test for independent significance of baseline characteristics and explanatory variables. The performance of relevant parameters was assessed by using the Kaplan–Meier method, and differences in survival between groups were compared by using the log-rank test. In addition, the receiver operating characteristic (ROC) curves of BUN/ALB for predicting 30-day mortality with HAP were plotted. Sensitivity of a score less than the cutoff point indicated 30-day mortality, while specificity of that greater than the cutoff point indicated survival beyond 30 days, both of which could be evaluated for each possible cutoff point. The cutoff point representing the highest Youden index (specificity + specificity − 1) was selected as the optimal threshold value. Hazard ratios (HRs) and 95% confidence intervals (CIs) were pooled to measure the effects of relevant parameters on prognosis. An HR greater than 1 indicated a worse prognosis in patients with a relevant parameter, while a HR less than 1 indicated a better prognosis. The criterion for statistical significance was set at an *α* of 0.05, and all *P* values were based on two-sided tests. Statistical analysis was performed using IBM SPSS Statistics, version 20 (IBM Corp, Armonk, NY, USA).

## 3. Results

**Figure 1:**
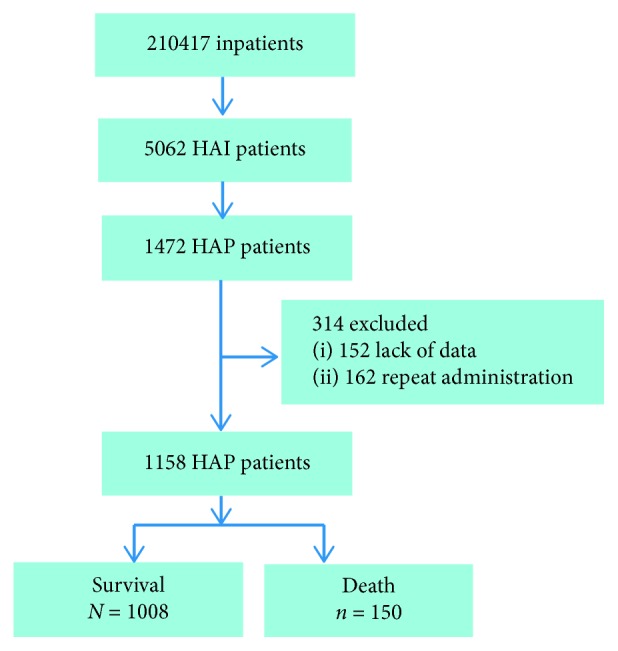
Analysis plan.

Among the 210,417 inpatients, 1472 (0.7%) cases of HAP were recorded. 314 of which were excluded because of missing key data or presence of repeated infection; finally, only a total of 1158 cases were included (Figure 1 and Table 1). Within 30 days, 150 (13.0%) patients died and 566 (48.9%) patients received antibiotic therapy during the last 90 days, while 30 (2.6%) patients were admitted in the ICU. A total of 327 (28.2%) patients had a blood lymphocyte count less than 0.8 × 10^9^/L and blood urea nitrogen/albumin (BUN/ALB) level of 0.21 ± 0.17, and 193 (16.7%) patients had MDR pathogens in their system (Table 2).

**Figure 2:**
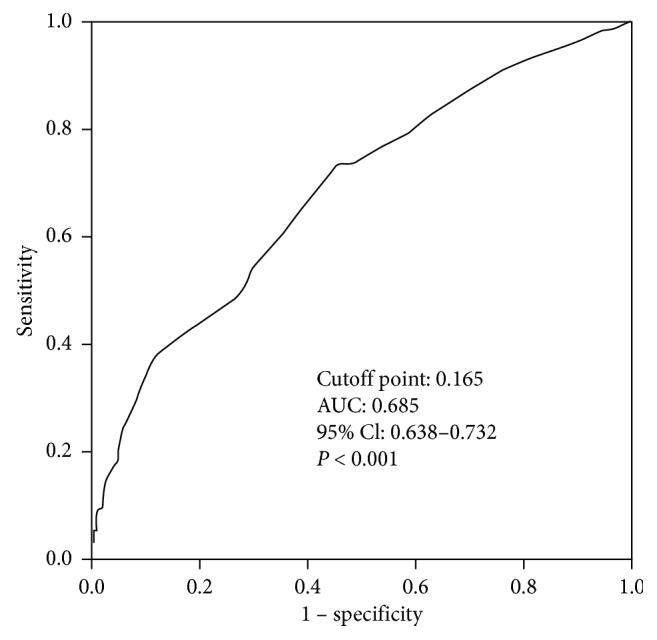
The ROC curve analysis of the BUN/ALB for predicting 30-day mortality with HAP. Abbreviation: ROC, receiver operating characteristic; AUC, area under the curve; CI, confidence interval; BUN/ALB: blood urea nitrogen/blood albumin; HAP: hospital-acquired pneumonia.

**Figure 3:**
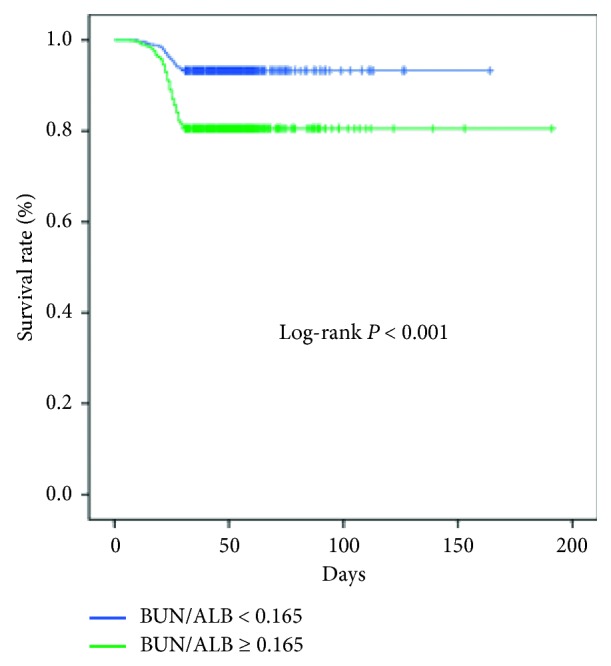
Kaplan–Meier curves of 30-day survival in HAP patients stratified by BUN/ALB. A high BUN/ALB was significantly associated with a worse survival than a low BUN/ALB (*P* < 0.001). HAP: hospital-acquired pneumonia; BUN/ALB: blood urea nitrogen/blood albumin.

The univariate Cox regression analysis identified the following independent factors that affect 30-day mortality with HAP (Table 3): age greater than 70 years, presence of diabetes mellitus and COPD, gastric tube intubation, administration of proton-pump inhibitor, blood albumin level less than 30 g/l, elevated neutrophil-to-lymphocyte ratio, antibiotics therapy in the preceding 90 days, ICU admission, blood lymphocyte count less than 0.8 × 10^9^/L, elevated BUN/ALB levels, and presence of MDR pathogens. Multivariable analysis revealed that administration of proton-pump inhibitor (OR = 1.508; 95% CI, 1.003–2.268; *P*=0.048), antibiotic therapy in the preceding 90 days (OR = 1.875; 95% CI, 1.278–2.751; *P*=0.001), ICU admission (OR = 2.405; 95% CI, 1.334–4.338; *P*=0.004), blood lymphocyte count less than 0.8 × 10^9^/L (OR = 1.626; 95% CI, 1.126–2.348; *P*=0.009), elevated BUN/ALB (OR = 3.871; 95% CI, 2.174–6.893; *P* < 0.001) levels, and presence of MDR pathogens (OR = 1.870; 95% CI, 1.252–2.794; *P*=0.002) were still associated with 30-day mortality. ROC analysis presented similar results with Cox regression and revealed that the BUN/ALB was a significant predictor of 30-day survival. The area under the ROC curves in the BUN/ALB predicting 30-day mortality with HAP was 0.685 (Figure 2), and the cutoff point was 0.165 with the highest predictive performance for both specificity and sensitivity (Figure 2). A high BUN/ALB level was significantly associated with worse survival than that of a low BUN/ALB (*P* < 0.001) (Figure 3).

## 4. Discussion

We revealed that the mean mortality rate in this study was 13.0%, consistent with the HAP mortality reported by other researchers [8, 14]. In the ICUs, the mortality was the highest, similar to that seen in the previous studies [8, 15] because the patients often had multiple organ dysfunction or underwent bronchoscopy [15]. Furthermore, we identified significant prognostic factors for 30-day mortality among HAP patients, including administration of proton-pump inhibitor, antibiotic therapy in the preceding 90 days, ICU admission, blood lymphocyte count less than 0.8 × 10^9^/L, elevated BUN/ALB levels, and presence of MDR pathogens.

As HAP is often associated with peptic disease and nonsteroidal drugs are usually used to allay patients' fever, the administration of proton-pump inhibitor was common in HAP patients. In the current study, we found that the administration of proton-pump inhibitor had a negative effect on HAP patients' 30-day mortality, consistent with the literature report [16]. Whether patients received antibiotic therapy in the preceding 90 days showed a significant difference in the outcomes of the present study, suggesting its importance as a prognostic factor for 30-day mortality with HAP. This finding is similar to that of previous studies that suggested the impact of virulence and antibiotic tolerance [17]. Meanwhile, antibiotic therapy in the preceding 90 days resulted in an increase of MDR pathogenesis, thereby leading to worse prognosis and higher mortality [18]. Presence of MDR pathogens had a negative effect on HAP patients' 30-day mortality in this study. It was similar to this study because MDR pathogens' virulence was stronger and the selection of antimicrobials was fewer [19]. In the current study, the 30-day mortality was still related to ICU admission because the illness maybe severe, implying the need for ICU admission [15], and antimicrobial resistance is an increasing concern in the ICUs worldwide [20]. Blood routine examination is common for patients, and the lymphocyte count is easy to calculate and very suitable for clinical application. Blood lymphocyte count less than 0.8 × 10^9^/L was also an independent risk factor for 30-day mortality in this study. It was similar to a previous study [21]. The BUN/ALB level is a simple but an independent predictor of mortality and severity of pneumonia [22]. An elevated BUN/ALB level is associated with higher 30-day mortality in the present study. Patients with pneumonia often had hydration status resulting in increasing reabsorption of urea by the kidneys, and elevation of BUN level is frequently observed [23]. It is interesting to note that earlier studies mostly focused on community-acquired pneumonia showed that nonsurvivors have significantly elevated BUN levels and lower serum albumin levels than those of survivors [24]. Our current study was one of the few researches highlighting BUN/ALB level as a predictor of 30-day mortality in a large sample. In addition, the cutoff point of BUN/ALB level was 0.165. A high BUN/ALB level was significantly associated with a worse survival than a low BUN/ALB. This will help clinicians to understand better what high BUN/ALB ratio means.

There are several limitations of our study that may decrease the impact of the results presented. First, as a single-center retrospective study, the assessment of HAP may contain bias. Second, there was no microbiologic documentation in half of the patients. It can be argued that some patients without etiologic diagnosis may not actually have had HAP. However, this is the usual situation in clinical practice, and our rate of etiologic diagnosis was similar to the ancillary reports in this field.

## 5. Conclusion

The purpose of the current study is to evaluate the factors that affect 30-day mortality of patients with HAP which have been highlighted in the findings. The results indicate that the mortality rates of HAP were different in each department and that peripheral blood BUN/ALB level is a simple but independent predictor of 30-day mortality with HAP. With solid statistics, the findings of this research are consistent with those of similar studies of prognostic factors of 30-day mortality with HAP.

## Figures and Tables

**Table 1:** Epidemiology and 30-day mortality of HAP in each department.

Department	Inpatient, *n*=210417 (%)	HAP, *n*=1158 (%)	Death, *n*=150 (%)	30-day mortality (%)
SICU	465 (0.22)	7 (0.60)	4 (2.7)	57.1
MICU	957 (0.45)	23 (1.99)	11 (7.3)	47.8
Gastroenterology	6366 (3.03)	22 (1.90)	9 (6.0)	40.9
Rheumatology	6479 (3.08)	23 (1.97)	8 (5.3)	34.8
Oncology	11706 (5.56)	79 (6.82)	25 (16.7)	31.6
Traditional Chinese medicine	3652 (1.74)	7 (0.60)	2 (1.3)	28.6
Respiratory medicine	5294 (2.51)	45 (3.87)	11 (7.3)	24.4
Infectious diseases	19651 (9.33)	99 (8.55)	20 (13.3)	20.2
Nephrology	4308 (2.04)	39 (3.37)	7 (4.7)	17.9
Gastrointestinal surgery	8326 (3.96)	42 (3.63)	6 (4.0)	14.3
Cardiovascular	9623 (4.57)	141 (12.18)	18 (12)	12.8
Invasive technology	8348 (3.97)	9 (0.78)	1 (0.7)	11.1
Spine surgery	3580 (1.70)	21 (1.81)	2 (1.3)	9.5
Neurology	12405 (5.90)	144 (12.44)	12 (8.0)	8.3
Endocrinology	9307 (4.42)	26 (10.79)	2 (1.3)	7.7
Neurosurgery	2886 (1.37)	57 (4.92)	4 (2.7)	7
Rehabilitation	4171 (1.98)	125 (10.79)	5 (3.3)	4
Hepatological surgery	12860 (6.11)	69 (5.96)	2 (1.3)	2.9
Psychiatry	11668 (5.55)	126 (10.88)	1 (0.7)	0.8
Joint surgery	5124 (2.44)	9 (0.78)	0	0
Urology	10853 (5.16)	17 (1.47)	0	0
Thyroid surgery	10766 (5.12)	13 (1.12)	0	0
ENT	10636 (5.05)	8 (0.69)	0	0
Dermatology	2979 (1.42)	1 (0.09)	0	0
Gynaecology and obstetrics	28007 (13.31)	6 (0.52)	0	0

HAP: hospital-acquired pneumonia; SICU: surgical intensive care unit; MICU: medical intensive care unit; ENT: ear, nose, and throat.

**Table 2:** Demographic, laboratory, and clinical variables of HAP.

Characteristics	Value
Age >70 years	409 (35.3%)
Gender: male	723 (62.4%)
Smoke	248 (21.4%)
Diabetes mellitus	230 (19.9%)
COPD	55 (4.7%)
Antibiotics therapy in the preceding 90 days	566 (48.9%)
Stomach tube intubation	307 (26.5%)
Central venous catheterization	197 (17.0%)
Use of PPI	745 (64.3%)
ICU admission	30 (2.6%)
ALB <30 g/L	124 (10.7%)
WBC, ×10^9^/L	10.44 ± 5.72
Lymphocyte count <0.8 ∗ 10^9^/L	327 (28.2%)
NLR	9.08 ± 9.06
BUN/ALB	0.21 ± 0.17
MDR pathogens	193 (16.7%)
Related mortality	150 (13.0%)

HAP: hospital-acquired pneumonia; COPD: chronic obstructive pulmonary disease; use of PPI: use of proton-pump inhibitor; ICU admission: intensive care unit admission; ALB: albumin; WBC: white blood cell; NLR: neutrophil-to-lymphocyte count ratio; BUN/ALB: blood urea nitrogen/blood albumin; MDR: multidrug resistant.

**Table 3:** Univariate and multivariate cox regression analyses of factors affecting 30-day mortality with HAP.

Characteristics	Related mortality, *n*=150 (13.0%)	Survival, *n*=1008 (87.0%)	Univariate			Multivariate		
			HR	95% Cl	*P*	HR	95% Cl	*P*
Age >70 years	71 (47.3%)	338 (32.5%)	1.701	1.234–2.343	0.001	1.329	0.943–1.874	0.105
Gender: male	103 (68.7%)	620 (61.5%)	1.344	0.952–1.898	0.093			
Smoke	33 (22.0%)	215 (21.3%)	1.045	0.710-1.537	0.825			
Diabetes mellitus	40 (26.7%)	190 (18.8%)	1.524	1.061–2.188	0.023	1.366	0.936–1.995	0.106
COPD	12 (8.0%)	43 (0.43%)	1.843	1.022–3.324	0.042	1.075	0.569–2.030	0.824
Antibiotics therapy in the preceding 90 days	106 (70.7%)	460 (45.6%)	2.684	1.888–3.815	<0.001	1.875	1.278–2.751	0.001
Stomach tube intubation	54 (36.0%)	253 (25.1%)	1.606	1.151–2.442	0.005	0.899	0.616–1.313	0.582
Central venous catheterization	32 (21.3%)	165 (16.4%)	1.348	0.912–1.992	0.134			
Use of PPI	118 (78.7%)	627 (62.2%)	2.138	1.447–3.161	<0.001	1.508	1.003–2.268	0.048
ICU admission	15 (10.0%)	15 (1.5%)	5.190	3.043–8.852	<0.001	2.405	1.334–4.338	0.004
ALB < 30 g/l	27 (18.0%)	97 (9.6%)	1.904	1.255–2.888	0.002	1.346	0.874–2.074	0.177
WBC, mean ± SD, ^∗^10^9^/L	10.17 ± 8.43	10.47 ± 5.19	0.991	0.961–1.023	0.586			
Lymphocyte count <0.8 × 10^9^/l	64 (42.7%)	263 (26.1%)	1.964	1.421–2.714	<0.001	1.626	1.126–2.348	0.009
NLR	11.18 ± 11.37	8.76 ± 8.62	1.019	1.007–1.031	0.002	1.004	0.987–1.022	0.619
BUN/ALB	0.33 ± 0.28	0.20 ± 0.15	9.100	5.647–14.663	<0.001	3.871	2.174–6.893	<0.001
MDR pathogens	51 (34.0%)	142 (14.1%)	2.835	2.022–3.975	<0.001	1.870	1.252–2.794	0.002

HAP: hospital-acquired pneumonia; COPD: chronic obstructive pulmonary disease; use of PPI: use of proton-pump inhibitor; ICU admission: intensive care unit admission; ALB: albumin; WBC: white blood cell; NLR: neutrophil-to-lymphocyte count ratio; BUN/ALB: blood urea nitrogen/blood albumin; MDR: multidrug resistant.

## Data Availability

The data used to support the findings of this study are available from the corresponding author upon request.
